# A modified self‐controlled case series method for event‐dependent exposures and high event‐related mortality, with application to COVID‐19 vaccine safety

**DOI:** 10.1002/sim.9325

**Published:** 2022-01-28

**Authors:** Yonas Ghebremichael‐Weldeselassie, Marie Joëlle Jabagi, Jérémie Botton, Marion Bertrand, Bérangère Baricault, Jérôme Drouin, Alain Weill, Mahmoud Zureik, Rosemary Dray‐Spira, Paddy Farrington

**Affiliations:** ^1^ School of Mathematics and Statistics The Open University Milton Keynes UK; ^2^ EPI‐PHARE French National Agency for Medicines and Health Products Safety (ANSM), French National Health Insurance (CNAM) Saint‐Denis France; ^3^ Faculté de Pharmacie Université Paris‐Saclay Châtenay‐Malabry France; ^4^ Anti‐infective Evasion and Pharmacoepidemiology Centre for Epidemiology and Population Health (CESP) Montigny‐le‐Bretonneux France

**Keywords:** cardiovascular events, COVID‐19, epidemiological methods, self‐controlled case series, vaccines

## Abstract

We propose a modified self‐controlled case series (SCCS) method to handle both event‐dependent exposures and high event‐related mortality. This development is motivated by an epidemiological study undertaken in France to quantify potential risks of cardiovascular events associated with COVID‐19 vaccines. Event‐dependence of vaccinations, and high event‐related mortality, are likely to arise in other SCCS studies of COVID‐19 vaccine safety. Using this case study and simulations to broaden its scope, we explore these features and the biases they may generate, implement the modified SCCS model, illustrate some of the properties of this model, and develop a new test for presence of a dose effect. The model we propose has wider application, notably when the event of interest is death.

## 1 INTRODUCTION

Safe and effective vaccination against SARS‐CoV‐2, achieving high coverage worldwide, is key to bringing the COVID‐19 pandemic to an end. The introduction of COVID‐19 vaccines in many countries has prompted several field investigations of their safety profile. This task is complicated by the fact that the vaccines were often targeted initially at individuals deemed to be at high risk owing to their age and underlying state of health. Vaccinees may also differ in other respects such as ethnicity, socioeconomic factors, and lifestyle. The unvaccinated population is thus not directly comparable to vaccinees at any point in time. In this context, the self‐controlled case series (SCCS) design can be an attractive option as it does not rely on between‐individual comparisons, but is self‐matched, and thus allows automatically for confounders that do not vary over time.^1^, ^2^ It has been used in several studies of COVID‐19 vaccine safety.^3^, ^4^, ^5^, ^6^

The present case study concerns a SCCS study of the Pfizer‐BioNTech vaccine against SARS‐CoV‐2 undertaken in France.^4^ This was a nationwide study of cardiovascular events and their potential relation to vaccination, based on all such events arising in December 2020 to April 2021. The paper is motivated, in the main, by two methodological issues which may arise together in SCCS studies of COVID‐19 vaccine safety. The first is that vaccination may be delayed or cancelled following occurrence of an adverse event. The second is that the adverse events of interest may increase short‐term mortality.

The suite of SCCS models currently available can deal with each of these issues separately. Thus, an SCCS model for event‐dependent exposures has been developed for use when events affect subsequent exposures (in our case, exposures are COVID‐19 vaccinations); but this assumes that events do not increase short‐term mortality.^7^ Conversely, an SCCS model has been developed for use with events that increase short‐term mortality; but it is not applicable when events also affect subsequent exposures (other than through death).^8^ A further complication is that COVID‐19 vaccines are typically administered in two‐dose schedules, with the doses only a few weeks apart.

However, it turns out that a simple adjustment to the SCCS model for event‐dependent exposures resolves these issues, provided that most of the postevent deaths observed are caused by the event. In fact, this modified SCCS model is also valid when the event of interest is death: this represents a significant extension of the applicability of SCCS methodology.

We address two further questions. The first is whether unvaccinated cases should be included in the analysis. Secondly, we propose a simple test to determine whether the vaccine has the same effect (or lack of effect) at different doses; this is nontrivial because the model we use is not likelihood‐based, and so a likelihood ratio test is unavailable.

In this paper we study these issues using both real and simulated data. The paper is organized as follows. In Section 2 we give some details of the SCCS method, its extension for event‐dependent exposures, and the SCCS model formulation and target parameter. Then in Section 3 we describe the French study, illustrate the methodological issues of interest, and detail our proposed solutions. In Section 4 we motivate, describe, and report on three sets of simulations. The paper ends with a discussion of the findings and of their likely applicability.

## 2 THE SCCS METHOD

The SCCS method is described in a *Statistics in Medicine* tutorial paper,^1^ and in much greater detail in a book on the topic.^2^

The standard SCCS model is obtained by conditioning on the number of events observed for each individual within a retrospective Poisson cohort model, the cohort having been followed over a specified observation period. Individuals with zero events do not contribute to the conditional likelihood, which includes only cases (whence the CS in SCCS). Individual likelihood contributions are of the form:

$$\frac{\lambda (t;x)}{\int_{a}^{b}\lambda (s;x)ds},$$

where (a,b] is the observation period, *t* is the event time, λ(s;x) is the event rate at time *s*, and *x* is the exposure history for this individual. This likelihood is valid for events with independent recurrences and (approximately) for rare nonrecurrent events. The ratio form of the conditional likelihood contribution implies that each case acts as its own control (when the SC in SCCS). Time‐invariant covariates acting multiplicatively on the event rate factor out of the likelihood: thus, multiplicative time‐invariant confounders are automatically adjusted (time‐varying confounders, on the other hand, are not).

However, for inferences about the event rate λ(s;x) from the conditional likelihood to be valid, some exogeneity conditions are required: neither postevent exposures nor the observation period (a,b] should depend on the event time *t*. These conditions may be violated if the event can cause subsequent vaccination to be delayed or cancelled, or if it causes an early death.

An extension of the standard SCCS model has been developed to handle situations in which occurrence of an event affects the timing or the occurrence of subsequent exposures.^2^, ^7^ In this model, all exposures that occur after an event are disregarded, and treated as missing: this is because their timing may be dependent on the event. The model is estimated using unbiased estimating equations rather than a likelihood. The estimating equations are derived under a counterfactual in which no exposures can occur after an event: thus, the timing of such exposures is immaterial and does not affect the estimation. Two conditions are required: postexposure risk periods must not be indefinite, and their duration must be known once they start. This is the model we shall adapt in the present paper.

In the SCCS models used in the present paper, the event rate is represented as piecewise constant, with parameters for exposure levels and time effects. The exposure levels are determined by risk intervals after each dose of COVID‐19 vaccine. Days outside the risk periods correspond to the reference exposure level. The models are loglinear with model equation of the form:

Exposure level + Seasonal effect.

The exposure parameters are log relative incidences log(ρ), where

$$\rho =\frac{\lambda (s;s\;\text{in risk period})}{\lambda (s;s\;\text{not in risk period})}.$$

Estimation of the SCCS model for event‐dependent exposures proceeds by iteratively reweighting observations in such a way that they conform to our counterfactual, and maximizing a Poisson pseudo‐likelihood. This is chosen so as to produce the correct estimating equations. The procedure yields consistent parameter estimates, their covariance matrix being obtained using a sandwich estimator.^2^

## 3 FRENCH STUDY OF COVID‐19 VACCINATION AND HEMORRHAGIC STROKE

The aim of the French study was to quantify the relative incidence of a range of cardiovascular events after vaccination with the Pfizer‐BioNTech vaccine (BNT 162b2 mRNA) against SARS‐CoV‐2 infection and COVID‐19 disease, using a SCCS design.^4^ We focus on one endpoint: hemorrhagic stroke.

The data were extracted from the French national health data system SNDS linked to the vaccination database VAC‐SI. The SNDS is a system of linked health‐related databases covering 99% of the French population (67 million people).^9^, ^10^, ^11^, ^12^ Hospital data from the PMSI database within SNDS comprises admission and discharge information, including diagnostic codes based on the 10th revision of the International Classification of Diseases and Related Health Problems (ICD10). A 2015 study showed that ICD10 codes for hemorrhagic stroke recorded in the PMSI database had a positive predictive value of 77%.^13^

The results reported here are based on a preliminary dataset of 2894 cases of haemorrhagic stroke, of whom 894 received at least one vaccine dose. These data were used for investigative purposes; the published study, which used updated data, included 3514 cases aged 75 years and older.^4^

The dataset used for the present study included all individuals with a first haemorrhagic stroke occurring within a 96‐day observation period stretching from December 15, 2020 to March 20, 2021. The postvaccination risk period of interest included days 1 to 14 after each of the two vaccine doses (day 0 was excluded for reasons that will be set out in Section 3.4). The event is sufficiently rare that the SCCS method may validly be applied.^14^ Further details of the analysis are given in Section 3.4.

All analyses reported here were undertaken in R.^15^ The SCCS models were fitted with the R package SCCS.^16^

### 3.1 Event‐dependent exposures

Of the 894 vaccinated cases, 407 had received both vaccine doses. The median interval between doses was 23 days, with minimum and maximum separations of 16 and 49 days, respectively.

**FIGURE 1:**
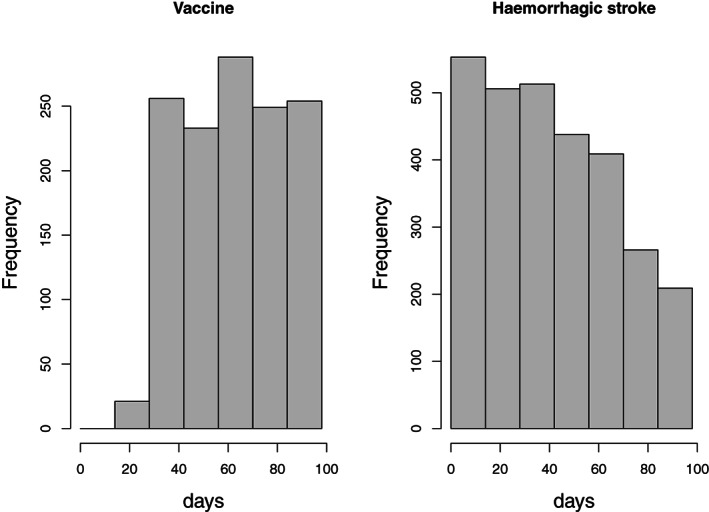
Left: temporal distribution of vaccinations (either dose). Right: temporal distribution of hospital admissions for haemorrhagic stroke. Day 0 is December 15, 2020

The distributions of vaccinations (both doses combined) and hemorrhagic strokes by calendar day since December 15, 2020 are shown in Figure 1. This shows a drop in the number of hemorrhagic strokes over time. This is due to the time required to verify the data and update the central SNDS database; these delays are unrelated to vaccination status or time since vaccination.

**FIGURE 2:**
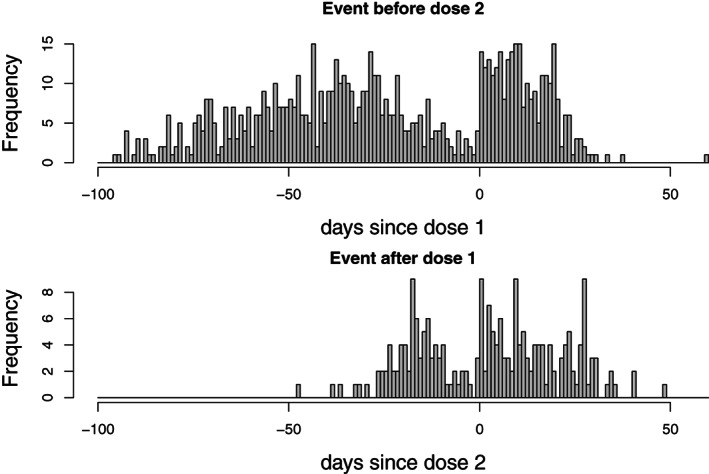
Days from COVID‐19 vaccination to hemorrhagic stroke. Top: days from dose 1, for events occurring before dose 2 if present, or at any time if dose 2 not present. Bottom: days from dose 2, for events occurring after dose 1

Histograms of the interval between vaccination and the event, for each dose, are shown in Figure 2. The top panel shows all time intervals from dose 1 to event, for events occurring before dose 2 if given (and for all events in cases who received dose 1 but not dose 2); the bottom panel shows the time intervals between dose 2 and the event, for all events occurring after dose 1. (Thus, events occurring between doses 1 and 2 feature in both panels.)

A striking feature of these histograms is the dip in the number of events occurring just before each dose (represented by day 0 in Figure 2). This dip occurs because individuals who have had a hemorrhagic stroke are likely to delay vaccination until they have recovered sufficiently, and may even subsequently avoid vaccination completely. This is an example of event‐dependence of exposure (the exposure being COVID‐19 vaccination). The dip before the first dose spans at least 40 days. Some individuals may, of course, delay vaccination beyond the end of observation, and indeed may postpone vaccination indefinitely. This more extreme form of event‐dependence of exposures cannot readily be detected from Figure 2.

Delaying or cancelling vaccination after an event has occurred will tend to inflate the relative incidence. When vaccination is only ever delayed by a short period, this bias may be corrected within the standard SCCS model by including a prevaccination risk period (the corresponding parameter represents a reverse causality effect of the event on vaccination).

To study the impact of such delays, we fitted standard SCCS models with prevaccination risk periods of increasing length; for simplicity we took the postvaccination risk period to be 1 to 14 days and assumed that the same effect, if any, was present at both doses. In these standard SCCS models we ignored any impact of deaths, and ended observation at time of death for cases who died. Our focus here is not on the relative incidences per se, but on how their estimates vary with the prevaccination risk period; deaths will be discussed in the next section. For each value of τ, the prevaccination risk interval stretches from 1 to τ days before each dose.

**FIGURE 3:**
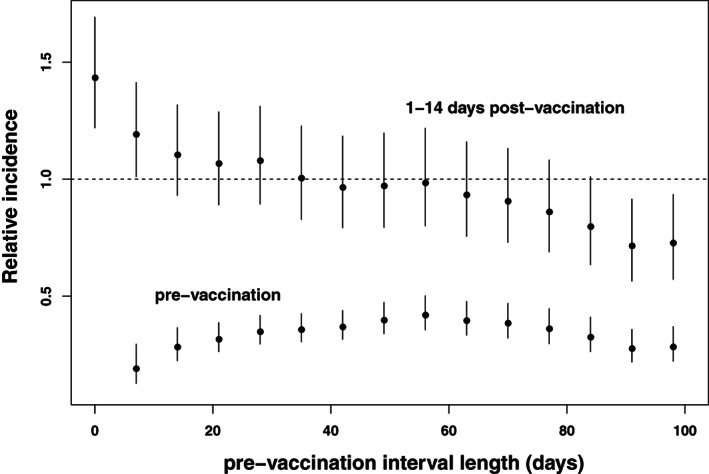
Relative incidence (with 95% confidence intervals) by duration τ of the prevaccination risk period. Top series: relative incidence in the 1 to 14 days postvaccination period; Bottom series: relative incidence in the period 1 to τ days prior to vaccination

The results are displayed in Figure 3. This shows that the relative incidences for the prevaccination risk periods are all significantly less than 1. The relative incidences for the 1 to 14 day postvaccination risk period declines nearly monotonically as τ increases. When no prevaccination risk period is included (τ = 0), Figure 3 indicates that the relative incidence is significantly elevated above 1; when all prevaccination time is included as a separate period (τ=98), the relative incidence is significantly reduced below 1.

If occurrence of an event led only to a brief delay in vaccination, the relative incidences for the 1‐ to 14‐day risk period would converge to a constant value as τ increases. The fact that they do not indicates that the delay in vaccination is not always brief. In consequence, it is not possible to correct for event‐dependence simply by including a prevaccination risk period. Instead, we shall use the SCCS model for event‐dependent exposures outlined in Section 2. In this model, it is assumed that, after an event has occurred, vaccination may be delayed or cancelled entirely, in a manner that need not be specified.

### 3.2 Deaths caused by the event

**FIGURE 4:**
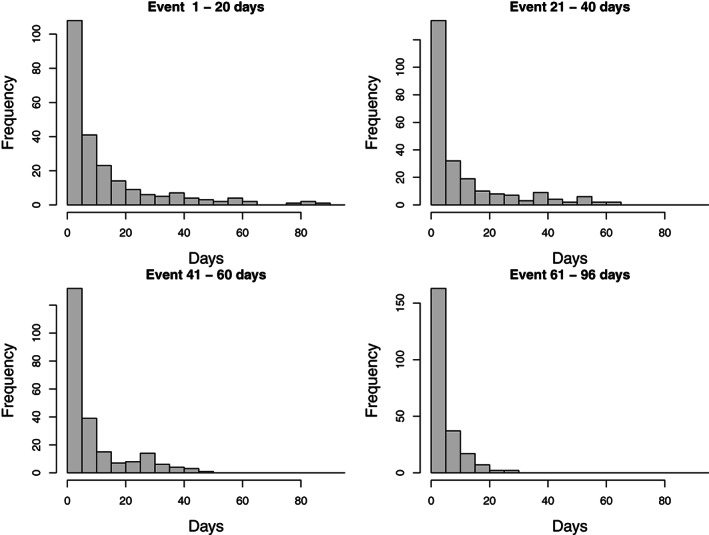
Days from event to death (5‐day bins) for individuals who died, stratified by quartile of day of event in those cases

Several field investigations of the safety of COVID‐19 vaccines have investigated serious adverse events that are associated with increased short‐term mortality. This is unusual in vaccine safety studies, which (with some exceptions) tend to deal with adverse events with few long‐term sequelae (a common example being febrile convulsions). Hemorrhagic stroke is a case in point, as it is associated with very high mortality: in our data, 927 of the 2894 cases (32%) died after the event during the relatively brief observation period (others died later). The median time from event to death, in those who died during the observation period, is 4 days. The distribution of times from event to death is shown in Figure 4.

Rather than presenting the time from event to death as a single histogram in Figure 4, we have stratified its distribution according to the quartile of calendar day of event (counted from 15 December 2020) for cases who died within the 96‐day observation period. This is because a short interval from event to death can simply reflect the fact that the event occurred late within the observation period. Thus, the distributions in Figure 4 are conditional on calendar day of event. The fact that all the four distributions have sharp modes close to zero suggests that most deaths were causally related to the event. If they were not, we would expect the distributions to flatten out at lower quartiles.

The duration of the short‐term excess mortality from haemorrhagic stroke may be estimated using a standard SCCS model for the 927 deaths. In this model, death is the event, hemorrhagic stroke is the exposure, and observation periods stretch from the day of the haemorrhagic stroke to the study end on March 20, 2021. This model was previously proposed for SCCS analyses for deaths after a single exposure.^17^ We used four 5‐day risk periods, and a temporal adjustment in two‐weekly intervals. The results are in Table 1.

**TABLE 1:** Relative incidence (RI) and 95% confidence interval (CI) for death after hemorrhagic stroke by risk period

Risk period (days)	Events	RI	95 % CI
Control period	134	1.00	
0 to 4 days	495	8.44	(5.76, 12.4)
5 to 9 days	169	3.44	(2.38, 4.96)
10 to 14 days	84	2.06	(1.43, 2.96)
15 to 19 days	45	1.31	(0.89, 1.94)

The results displayed in Table 1 strongly suggest that hemorrhagic stroke induces high short‐term mortality up to 14 days after the event.

In standard SCCS models, deaths that are caused by the event complicate the analysis. Deaths censor the observation period, which is conditioned upon in the analysis, but because the deaths are caused by the event, they are informative about the timing of the event. The model may need to be adjusted to allow for this, and a special SCCS model has been developed for this purpose.^8^ However, this model does not cater for event‐dependent exposures of the type described in the previous section. Also, the model does not apply when the event of interest is death: it is designed for events other than death that increase the short‐term mortality rate.

However—and this is the key insight of the present paper—the SCCS model for event‐dependent exposures can be used when all deaths are due to the event (and thus also when the event is death): we simply use the planned end of observation as the actual end of observation for each case, rather than date of death. The planned end of observation is the last time at which the event could have occurred and been ascertained. It is usually determined by the time limits that define the case ascertainment procedure; here it corresponds to March 20, 2021.

The rationale for this modification stems from the fact that, in the SCCS model for event‐dependent exposures, deaths due to the event of interest have no impact on the estimation procedure. The model is based on a counterfactual in which exposures can never occur after an event. Thus, deaths after an event do not censor exposures, since there are none in our counterfactual. In addition, deaths due to the event do not constrain possible event times, because if the event had not occurred, neither would the death. Hence, observation periods for cases who die of the event should not end at death (which is informative about the event time), but at the planned end of observation (which is not). In contrast, deaths not caused by the event do constrain the observation of events: had the event not occurred, the case would still have died. For such cases, the observation period should end at death.

As noted above, using the planned end of observation (rather than date of death) was previously proposed for SCCS analyses for deaths.^17^ This model, however, was limited to unique exposures. Our present proposal is not: it may be used when there are successive exposures of predetermined duration, as is the case with multidose vaccines.

In practice, in circumstances in which the event is known to carry high mortality, but it is not known for certain in individual cases whether the event contributed to the case's death, we recommend proceeding as if all deaths were caused by the event: thus, all observation periods should be set equal to the planned end of observation. The validity of this assumption may then be investigated in sensitivity analyses, as described in Section 3.4.

In contrast, if the event is not known to be associated with an increase in short‐term mortality, then all observation periods should end at the earliest of death and planned end of observation, as in other SCCS studies. This also applies to other types of censoring completely at random unconnected with mortality (e.g., end of the database record).

On the other hand, if it is known which deaths are caused by the event and which deaths are not, then observation periods should end at the planned end of observation for cases who die due to the event, and at earliest of death and planned end of observation for deaths not due to the event.

This last approach may also be explored when the duration *D* of short‐term mortality due to the event is known or may be estimated (as in Table 1). Deaths occurring less than *D* days after the event are then nominally attributed to the event, and deaths occurring *D* or more days after are attributed to other causes.

### 3.3 Test for a dose effect

The SCCS model for event‐dependent exposures (with or without the modification for event‐related mortality described in Section 3.2) is not based on a likelihood, so the likelihood ratio test is not available formally to test the hypothesis that the vaccine effect is the same at both doses. Instead, when there are two doses, as is the case with our data, an approximate Wald significance test may be conducted using the test statistic

$$X^{2}=\frac{{\left({\hat{\theta }}_{1}-{\hat{\theta }}_{2}\right)}^{2}}{\mathrm{var}({\hat{\theta }}_{1})-2\text{cov}({\hat{\theta }}_{1},{\hat{\theta }}_{2})+\mathrm{var}({\hat{\theta }}_{2})},$$

which in large samples has, approximately, a chi‐square distribution on one degree of freedom under the null hypothesis $\theta_{1}=\theta_{2}$. Here, $\theta_{1}$ and $\theta_{2}$ are the log relative incidences for doses 1 and 2, respectively; the terms var and covar in the denominator denote estimates of the variances and covariance of the estimated parameters ${\hat{\theta }}_{1}$ and ${\hat{\theta }}_{2}$. The performance of this test will be investigated in Section 4.3.

In version 1.4 of the R package SCCS, an estimate of this covariance matrix may be accessed after fitting the SCCS model for event‐dependent exposures.^16^ The R code for this, together with a more general version of the test, applicable when there are more than two doses (or more than one risk period per dose), are described in Appendix.

### 3.4 Data analysis

In this section we apply these analysis methods to the French data on COVID‐19 vaccination and hemorrhagic stroke. We describe a suitable sensitivity study, and test for a dose effect.

In all models, including the standard SCCS models reported in earlier sections, the risk period of interest is the 1‐ to 14‐day risk period after each dose of vaccine. We also specified day 0 as a separate risk period: this was to ensure that it is not included in the baseline. Day 0 is excluded from the risk period of primary interest to avoid introducing bias. We do not know when during the day the vaccine was given, and thus cannot accurately calculate the time at risk on day 0. Also, we do not know which came first: vaccination, or the event (though it seems extremely unlikely that vaccination would take place immediately after a patient had suffered a haemorrhagic stroke). Note that the rationale for excluding day 0 from the risk period is quite different from that pertaining to analyses of COVID‐19 infection, where testing for infection on admission to hospital can induce spuriously large day zero effects.^18^

To account for possible seasonal variation in the baseline incidence of haemorrhagic stroke, temporal effects were adjusted in six 14‐day periods and a final 12‐day period. All events are used for this adjustment, including those arising in unvaccinated cases. Similar results were obtained with weekly rather than fortnightly intervals.

As discussed in Section 3.1, we used the SCCS model for event‐dependent exposures. When using this model, unlike the standard SCCS model, it is essential to include all cases, including those who are unvaccinated (this is discussed further in Section 4.2). We thus included all 2894 cases.

We fitted two SCCS models, based on the discussion of mortality in section 3.2. In the first model (Model 1), we defined the end of observation to be the end of case ascertainment on March 20, 2021, irrespective of whether the case had died. In the second model (Model 2), observation ended on March 20, 2021 if the death occurred less than D=15 days after the event, and at death if this occurred 15 or more days after the event; the value D=15 days was chosen based on Table 1. The results for Models 1 and 2 are in Table 2.

**TABLE 2:** Relative incidences (RI) and 95% confidence intervals (CI) for hemorrhagic stroke by risk period, for two self‐controlled case series models

		Model 1		Model 2	
Risk period (days)	Events	RI	95 % CI	RI	95% CI
Control period	2657	1.00		1.00	
Dose 1:					
Day 0	4	0.31	(0.11, 0.86)	0.31	(0.11, 0.86)
Days 1 to 14	166	1.09	(0.87, 1.36)	1.10	(0.88, 1.36)
Dose 2:					
Day 0	3	0.49	(0.16, 1.58)	0.50	(0.16, 1.61)
Days 1 to 14	64	0.93	(0.66, 1.33)	0.94	(0.66, 1.34)
Both doses:					
Day 0	7	0.38	(0.18, 0.82)	0.38	(0.18, 0.83)
Days 1 to 14	230	1.07	(0.86, 1.33)	1.07	(0.86, 1.33)

The results for the two models are virtually identical. Not unexpectedly, there is a statistically significant deficit of cases on day zero. In the 1‐ to 14‐day period, there is little suggestion of any effect at either dose.

The test described in Section 3.3, applied to Model 1, gives $X^{2}=0.7952,P=.37$. Thus, there is little evidence of a dose effect, and the estimate for both doses combined may be used to summarise the strength of association: *RI* =1.07 with 95% CI (0.86, 1.33).

Model 1 in Table 2 is valid under the assumption that all deaths are caused by the event, and thus impose no additional constraints on the observation periods. To study the sensitivity of the results to this assumption, we fitted a sequence of SCCS models, for each value *D* from the minimum to the maximum plus one number of days between event and death. For each such *D*, we assumed that all deaths with an interval d≥D between the event and death were not due to the event. For the corresponding cases, we ended the observation period at time of death, rather than at the end of case ascertainment, as described at the end of Section 3.2. In our dataset, time from event to death ranged from 0 to 87 days. With D=0 days, all deaths were presumed not to be due to the event, and so all observation periods were censored at death. With D=87+1=88 days, all deaths were presumed to be due to the event, so none were censored at death (this is Model 1 in Table 2).

**FIGURE 5:**
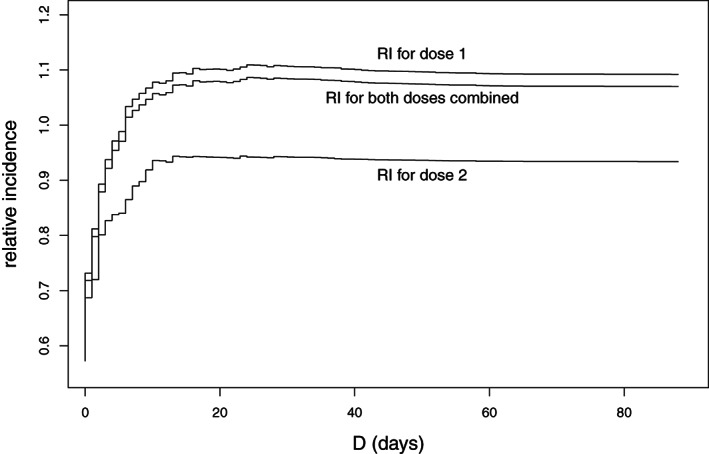
Relative incidence for the 1‐ to 14‐day risk period after doses 1 and 2, and both doses combined, for each value of *D* between 0 and 88 days. For each such *D*, deaths occurring *D* or more days after the event are assumed not to be due to the event

Figure 5 shows the estimated relative incidences obtained in this sequence of model fits. With D=88 days, the relative incidences in Figure 5 are those for Model 1 in Table 2. With D=15 days, the relative incidences correspond to those for Model 2 in Table 2. With D=0 they are very much lower: for both doses combined, *RI* =0.57; the 95% CI is (0.43, 0.77) (confidence intervals are not shown in Figure 5). However, the relative incidences plotted in Figure 5 rise very sharply with *D*. For *D* greater than or equal to 8 days, all three estimates are within 5% of the values in Table 2. The maximum RI values arise at day D=25 or 26. These maxima are *RI* =1.11 for dose 1, with 95% CI (0.89, 1.38); 0.94 (0.66, 1.34) for dose 2; and 1.09 (0.87, 1.35) for both doses combined. In these data, most deaths occurring within 14 days of the event are likely due to it, which provided the rationale for Model 2 of Table 2. We conclude that the results in Table 2 are robust.

## 4 SIMULATION STUDIES

In Section 4.1 we describe further investigations of the robustness of the model to failure of the assumption that all deaths are due to the event. In Section 4.2, we investigate the need to include all cases, not just vaccinated cases, when undertaking an analysis with the SCCS model for event‐dependent exposures. We also study the relative efficiency of the proposed model compared to the standard SCCS model, when event‐dependence of exposures is not an issue. Finally, in Section 4.3 we study the power of the test described in Section 3.3 for detecting a true dose effect. All these investigations were undertaken using simulations. The R code for the simulations is available from the journal website. All SCCS models fitted in the simulations used a nominal observation period of 100 days, risk periods 0 to 14 days after each vaccine dose (there is no day 0 issue as vaccinations occur as the day begins), and no temporal effects. However, the models we fitted did include temporal effects in fortnightly intervals, as generally recommended in SCCS analyses.

### 4.1 Robustness to the occurrence of deaths not due to the event

In this set of simulations, we sought to investigate the robustness of the proposed model to the presence of deaths not attributable to the event. Unlike the sensitivity study illustrated in Figure 5, which it complements, this investigation is not based on intervals from event to death, but on the proportions of cases who died for reasons unconnected with the event.

For a given proportion *p* of N=1000 cases, we assumed that the case died before the end of the observation period for reasons unrelated to the event. So, for example, if P=0.1 then, among all 1000 cases, 100 died for reasons unrelated to the event (irrespective of the number of deaths that are related to the event). These deaths were simulated as uniformly distributed within the planned observation period 1 to 100 days: thus they were generated completely at random. Planned vaccinations were independently simulated: first doses as uniformly distributed between 1 and 100 days, and second doses as uniformly distributed in an interval between 21 and 56 days after the first dose. First and second doses planned after date of death, and second doses planned after day 100, were therefore missing in the analysis. Finally, events were simulated using a multinomial distribution of index 1 for each case. For the Np cases dying completely at random, the multinomial distribution was supported on days 1 to death. For the remainder N(1−p) cases the multinomial distribution was supported on days 1 to 100. The daily multinomial probabilities were proportional to the vaccine‐related relative incidence ρ on days 0 to 14 after each vaccine dose, and proportional to 1 on other days within the support.

Note that the number of deaths due to the event is not specified: this could range from 0 to N(1−p) and is immaterial, as these deaths do not influence the event time or the analysis, as explained in Section 3.2. Only the Np deaths not due to the event have any impact. For each combination of p=0%,10%,20%,30% and ρ=1,2,3,4 we generated S=1000 simulation runs. For each of these runs, the dates of death, vaccination, and event were resampled, keeping only *p* and ρ fixed. We fitted the SCCS model for event‐dependent exposures, modified for event‐related deaths by ending all observation periods at day 100, the planned end of observation. The fact that for the Np cases who died at random the true observation period ends at day of death, rather than on day 100, may introduce bias, the study of which is the purpose of these simulations. We calculated the bias and the mean squared error (MSE) for the estimated parameter θ=log(ρ) as follows:

$$\begin{array}{ll} \text{Bias} & =\frac{1}{S}\overset{S}{\underset{i=1}{\sum }}\log({\hat{\rho }}_{i})-\log(\rho ),\;\\ \text{MSE} & =\frac{1}{S}\overset{S}{\underset{i=1}{\sum }}{\left(\log({\hat{\rho }}_{i})-\log(\rho )\right)}^{2}.\; \end{array}$$

We fitted two sets of models: vaccine effects at doses 1 and 2 estimated separately, and combined. We also evaluated the Monte Carlo SEs for both bias and MSE. The results for the combined dose 1 and dose 2 models are in Table 3.

**TABLE 3:** Bias and mean squared error (MSE) of the log relative incidence θ=log(ρ) (Monte Carlo SE) when a proportion *p* of cases die of causes unrelated to the event, for selected values of *p* and ρ

Proportion *p*	ρ=1	ρ=2	ρ=3	ρ=4
p=0				
Bias	−0.0021 (0.0028)	−0.0010 (0.0028)	0.0023 (0.0028)	0.0020 (0.0028)
MSE	0.0079 (0.0004)	0.0076 (0.0003)	0.0077 (0.0003)	0.0076 (0.0004)
p=0.1				
Bias	−0.0045 (0.0030)	−0.0148 (0.0028)	−0.0219 (0.0027)	−0.0268 (0.0028)
MSE	0.0089 (0.0004)	0.0081 (0.0004)	0.0077 (0.0004)	0.0086 (0.0004)
p=0.2				
Bias	−0.0072 (0.0030)	−0.0244 (0.0028)	−0.0302 (0.0028)	−0.0496 (0.0028)
MSE	0.0088 (0.0004)	0.0085 (0.0004)	0.0085 (0.0004)	0.0102 (0.0004)
p=0.3				
Bias	−0.0076 (0.0031)	−0.0285 (0.0028)	−0.0526 (0.0029)	−0.0651 (0.0030)
MSE	0.0096 (0.0004)	0.0085 (0.0004)	0.0110 (0.0005)	0.0133 (0.0005)

Table 3 shows that, when p=0%, so that no deaths are due to causes other than the event, the estimates are virtually unbiased (being less than two Monte Carlo SEs away from zero). When p>0%, there is a negative bias that increases in absolute value as *p* increases and also as ρ increases. However, even when *p* reaches the unlikely high value of 30%, the bias remains small, and the MSE is only slightly increased. For example, a bias of −0.02 means that a true relative incidence of 2 is estimated on average as 2exp(−0.02)=1.96. The results for each dose estimated separately are in Tables S1 and S2. The results for dose 1 are similar to those for the combined estimates in Table 2; for dose 2 the bias is less in absolute value than for dose 1, though the MSEs are higher than at dose 1.

We also investigated the even more extreme (and unlikely) situation in which p=0.9, in order to provide a bound for the likely bias. When ρ=1, the bias is then 0.0037 (MC SE 0.0036) for both doses combined. It increases in absolute value with ρ, and is −0.1004 (0.0039) when ρ=4. Even in this very extreme scenario, the bias is moderate.

### 4.2 Inclusion of unvaccinated cases and relative efficiency

In the standard SCCS model, inclusion of unvaccinated (or, more generally, unexposed) cases is not essential, as such cases do not contribute directly to the estimation of the vaccine effect. Such cases contribute primarily to the estimation of age or seasonal effects. Nevertheless, their inclusion is recommended when risk periods are long and there is potential for confounding between time since vaccination and age or season.

Matters are quite different when it comes to the SCCS model for event‐dependent exposures: for this model, it is necessary to include all or a random sample of unvaccinated cases. This is because lack of vaccination may indicate cancellation of vaccination, and may tend to occur more often for events that occur earlier (before they had the opportunity to be vaccinated). Thus, absence of vaccination may be informative about the timing of the event, and excluding unvaccinated cases may therefore introduce bias.

This may be explored using the haemorrhagic stroke data, by varying the number of unvaccinated cases included in Model 1 of Table 2. Table 4 shows the results of including no unvaccinated cases, of including a (randomly selected) half of them, and (for ease of comparison) of including all of them (as in Table 2).

**TABLE 4:** Impact of excluding unvaccinated cases on the relative incidence (RI) and 95% confidence interval (CI), for the 1‐ to 14‐day risk period

Number unvaccinated (% included)	0 (0%)	1000 (50%)	2000 (100%)
	RI (95% CI)	RI (95% CI)	RI (95% CI)
Dose 1	1.19 (0.96, 1.47)	1.11 (0.89, 1.38)	1.09 (0.87, 1.36)
Dose 2	0.96 (0.68, 1.36)	0.93 (0.66, 1.32)	0.93 (0.66, 1.33)
Both doses	1.15 (0.94, 1.41)	1.08 (0.88, 1.34)	1.07 (0.86, 1.33)

For the hemorrhagic stroke data, the impact of excluding unvaccinated cases is to bias the relative incidences upwards. The bias, in this case, is moderate. This is because the data also contain an appreciable proportion of vaccinated cases (498 of 894) for whom the event occurs before the first vaccine dose. These cases also contribute to the estimation of the temporal effect, but not (directly) to the estimation of the vaccine effect. Excluding cases with prevaccination events as well as all unvaccinated cases yields *RI* =1.74, with 95% CI (1.36,2.23).

However, no such vaccinated cases with prevaccination events will be available if all vaccinations are cancelled after an event has occurred (which arises when the event is a contraindication for vaccination). In these circumstances, the estimates may be much more severely biased if unvaccinated cases are excluded. The purpose of the following simulations is to demonstrate this point. For simplicity, no deaths due to causes other than the event are involved.

The simulation set‐up was the same as in Section 4.1 with p=0% (no deaths due to causes other than the event). For each run, we fitted two SCCS models for event‐dependent exposures: the first was fitted to the whole simulated dataset of 1000 cases. The second was fitted only to those cases in which the event followed a planned vaccination. Thus, events prior to vaccination were excluded in this second analysis: for all such events, it was assumed that any planned subsequent vaccinations were cancelled; these cases are therefore unvaccinated. Note that these cases were included and treated as unvaccinated in the first model, since in all these SCCS models, any postevent exposures are treated as missing.

The number of cases included in the analysis for the second model varied between simulation runs. However, the number of vaccinated cases was the same for the two models within each run. As in the previous section, the bias and MSE of θ=log(ρ) were obtained for values ρ=1,2,3,4, for each of the two vaccine doses and for both doses combined. The results for both doses combined are reported in Table 5.

**TABLE 5:** Timing of events (median number of cases, with interquartile range), bias and mean squared error (MSE) of the log relative incidence θ=log(ρ) (Monte Carlo SE) for models including all cases and for models including only cases with events postvaccination, for selected values of ρ

Data and model	ρ=1	ρ=2	ρ=3	ρ=4
Timing of events				
Event before dose 1	495 (484, 506)	422 (412, 433)	371 (361, 380)	332 (322, 343)
After dose 1 but before dose 2	309 (299, 318)	363 (353, 373)	403 (392, 413)	433 (421, 444)
All cases included				
Bias	0.0014 (0.0029)	0.0054 (0.0027)	0.0020 (0.0027)	0.0018 (0.0028)
MSE	0.0082 (0.0004)	0.0073 (0.0003)	0.0071 (0.0003)	0.0077 (0.0003)
Postvaccination cases only				
Bias	0.4659 (0.0035)	0.5133 (0.0034)	0.5300 (0.0035)	0.5451 (0.0036)
MSE	0.2291 (0.0033)	0.2750 (0.0036)	0.2930 (0.0038)	0.3099 (0.0039)

Table 5 shows that virtually unbiased estimates (the bias being within two Monte Carlo SEs of zero) were obtained when all cases are included, but not when only vaccinees were included in the analysis. In this latter scenario, the bias was large, positive, and increased with ρ; the MSEs were dominated by the bias term. For example, a bias of 0.5 means that a true relative incidence of 2 is estimated on average as 2exp(0.5)=3.3. The results for the separate doses are reported in Tables S3 and S4; when only vaccinees are included in the analysis, the bias is larger in absolute value at dose 1 than at dose 2.

In the simulations just described, we also fitted a third model: this was a standard SCCS model with observation periods ending on March 20, 2021. This model applies when event‐dependence of exposures is not an issue and there are no deaths. Comparing the MSEs with those obtained using the proposed model (that with all cases included, reported in Table 5) enables us to calculate the relative efficiency, both models producing virtually unbiased estimates:

$$\text{Relative efficiency}=\frac{\text{MSE}\;\text{for standard model}}{\text{MSE}\;\text{for proposed model}}\times 100.$$

The relative efficiencies at each dose and for both doses combined are shown in Table 6. This shows that there is a loss of efficiency, the relative efficiency being less than 100%, if the proposed model is used when there is no need for it, that is, when exposures are not event‐dependent and there are no event‐related deaths. The efficiency loss is greater at dose 2 than at dose 1, and increases with ρ.

**TABLE 6:** Relative efficiency (%) of estimation of the log relative incidence θ=log(ρ) for the proposed model compared to the standard self‐controlled case series model in the absence of event‐dependent exposure or event‐related deaths, by dose, for selected values of ρ

Dose	ρ=1	ρ=2	ρ=3	ρ=4
Dose 1	82.7	68.6	64.1	63.2
Dose 2	70.5	59.3	59.5	52.6
Both doses combined	77.0	66.2	63.0	62.8

The reduced efficiency stems from the fact that the SCCS model for event‐dependent exposures makes no use of available data on postevent exposures. Not using this information is justifiable in order to avoid bias when postevent exposures are influenced by the event. But when there is no such event‐dependence, not using these exposures will result in higher variances and wider confidence intervals.

### 4.3 Power to detect a true dose effect

The final set of simulations was undertaken to study the power of the test to detect a true dose effect, that is, a true difference between the vaccine effects at doses 1 and 2. We investigated two scenarios: no effect at dose 1, vs an effect at dose 2; and no effect at dose 2, vs an effect at dose 1.

**FIGURE 6:**
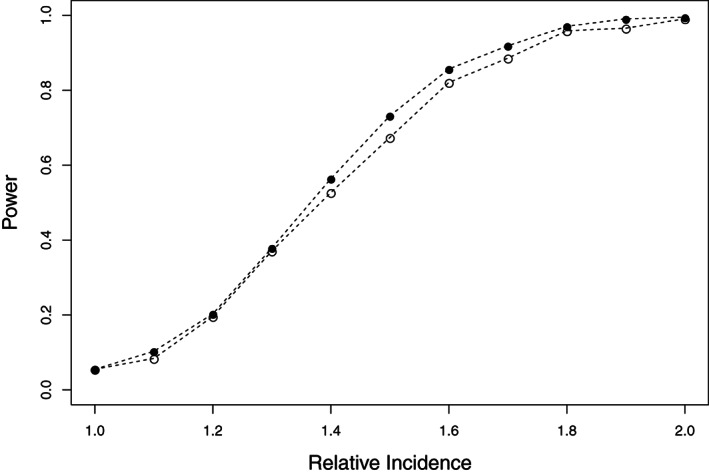
Empirical power of the test of the null hypothesis of no dose effect. Black dots: power with first dose effect $\rho_{1}=1$ and second dose effect $\rho_{2}$ equal to the relative incidence along the horizontal axis. Circles: power with second dose effect $\rho_{2}=1$ and first dose effect $\rho_{1}$ equal to the relative incidence along the horizontal axis

We used a similar setup to that previously described, with a fixed number of cases N=1000, again ignoring deaths not due to the event. The only difference with the simulations described previously was the specification of the relative incidences $\rho_{1}$ and $\rho_{2}$ at doses 1 and 2, respectively, which could differ. For each of S=1000 simulation runs (S=2000 for $\rho_{1}=\rho_{2}=1$), we fitted the SCCS model for event‐dependent exposures with separate dose effects, carried out the Wald test described in Section 3.3 with significance level 5%, and obtained the empirical size and power of the test of the null hypothesis that the vaccine effect is the same at the two doses. The results are shown in Figure 6.

The empirical size of the test (with $\rho_{1}=\rho_{2}=1)$ was 5.5%. This is a little higher than the nominal 5% significance level, though consistent with it (Monte Carlo SE 0.0051). Figure 6 shows that the test has reasonably good power in this scenario and with this sample size: the power to detect a dose effect is over 80% when the true dose‐specific relative incidences are 1 and 1.6 in either dose order.

## 5 DISCUSSION

Our main purpose in writing this paper was to propose a SCCS method for the analysis of COVID‐19 vaccine safety data for adverse events with high short‐term mortality, when occurrence of an event can preclude or severely delay subsequent vaccination. The solution we propose is to apply the SCCS model for event‐dependent exposures, with observation periods ending at the planned end of observation. This model is valid when all deaths are caused by the event. We have shown by simulations that it is robust to departures from this assumption, that is, when a proportion of cases die of causes unrelated to vaccination. In our simulations, this typically led to a small underestimation of the relative incidence.

Furthermore, the case study suggests that sensitivity to cause of death is greatest for cases who die within a few days of the event; these deaths are those most likely to be caused by the event. Thus, we have proposed an alternative approach, applicable when there is a clear excess of deaths shortly after the event. This involves censoring at death those individuals who die after that immediate post‐event period, but not those dying within it. The duration of this period may be chosen using a standard SCCS model for deaths, with observation periods starting at event.^17^

As with all statistical models, departure for assumptions may lead to incorrect inferences, and due consideration must be given to this possibility. To this end, sensitivity studies should be undertaken, which may include some of those suggested in the present paper.

These methods apply when cause of death is not known with certainty, which is usually the case. If cause of death is known, then cases who die of causes unconnected with the event should have their observation periods curtailed at date of death, whereas cases who die of the event should have their observation periods continued to the end of planned observation.

Our modified SCCS method is also valid when the event of interest is death, even when there are two or more successive exposures. Hitherto, such applications lay beyond the scope of the SCCS method. The modification we propose thus expands the range of its possible applications.

In addition to assumptions regarding cause of death, the other underlying assumptions required for the SCCS model for event‐dependent exposures are as follows. The event of interest must be nonrecurrent and rare; if the event is recurrent, the method should be applied to first occurrences. The risk period associated with the exposure must not be indefinite, and its duration must be known once it starts (these conditions are not met for some nonvaccine drug exposures). A further limitation is that concomitant time‐varying risk factors, other than age or season, cannot be adjusted in the current version of the model. For example, in an application to COVID‐19 vaccines, it might be advisable to control for SARS‐CoV‐2 infection (assuming that the data were available), if the infection were known to be a cause of the event of interest. In standard SCCS models this is possible; for the SCCS models considered here it is not.

If exposures are not event‐dependent, or if event‐dependence may be accommodated with a short pre‐exposure risk period, then the modified SCCS model we propose may not be optimal. Instead, the standard SCCS model (perhaps with adjustment for event‐related mortality^8^) may be preferable. On the other hand, avoidance of small biases may be more important than lower efficiency if very large amounts of data are available.

We also addressed two other issues of broad relevance to studies of vaccine safety using the SCCS model for event‐dependent exposures: inclusion of unvaccinated cases, and evaluation of dose effects. We showed that unvaccinated cases (or a random sample of them if they are very numerous) should be included in the analysis in order to avoid bias when vaccination may be cancelled after an event. We also proposed a new significance test to investigate dose effects, and provided simulation evidence of its good properties.

The methods described in this paper were developed specifically for COVID‐19 vaccines. It is unusual in vaccine safety studies to focus on adverse events with high short‐term mortality: typically, the events of primary interest tend not to carry serious long‐term health consequences. Nevertheless, the methods are applicable more widely should the need arise, both to vaccines other than COVID‐19 vaccines when serious adverse events, including death, are of concern, and to other applications in pharmacoepidemiology.

## Supporting information

Table S1. Bias and mean squared error of the log relative incidence θ=log(ρ) at dose 1 (Monte Carlo standard error) when a proportion *p* of cases die of causes unrelated to the event, for selected values of *p* and ρ.
Table S2. Bias and mean squared error of the log relative incidence θ=log(ρ) at dose 2 (Monte Carlo standard error) when a proportion *p* of cases die of causes unrelated to the event, for selected values of *p* and ρ.
Table S3. Bias and mean squared error of the log relative incidence θ=log(ρ) at dose 1 (Monte Carlo standard error) for models including all cases and for models including only cases with events post‐vaccination, for selected values of ρ.
Table S4. Bias and mean squared error of the log relative incidence θ=log(ρ) at dose 2 (Monte Carlo standard error) for models including all cases and for models including only cases with events post‐vaccination, for selected values of ρ.

Supporting Information

Supporting Information

## Data Availability

The licensing conditions under which the data were obtained preclude data sharing. We have therefore prepared a simulated data set that mimics key features of the original data. This dataset, and the R code to analyze it and run the simulations described in the paper, are freely available from the journal website.

## APPENDIX A

### A.1

We briefly set out how the test described in Section 3.3 may be extended to situations in which there are more than two doses, or more than one parameter per dose, and how it is performed in R. We start with the situation in which there are *k* doses, and it is required to test whether the relative incidences $\rho_{1},\rho_{2},...,\rho_{k}$, corresponding to equivalent risk periods after each of the *k* doses, are the same. The null hypothesis is thus $\theta_{1}=\theta_{2}=...=\theta_{k}$, where $\theta_{j}=\log(\rho_{j})$.

We reformulate this null hypothesis as k−1 separate linear hypotheses $\theta_{1}-\theta_{j}=0$, j=2,...,k. This reformulation is not unique; we have chosen to use dose 1 as reference because, in the SCCS model for event‐dependent exposures, dose 1 is likely to have more events associated with it than subsequent doses. Thus, the null hypothesis may be written as

Hθ=0,

where $\theta ={\left(\theta_{1},...,\theta_{k}\right)}^{T}$ and H is the following (k−1)×k matrix of linear contrasts:

$$\text{H}=\left(\begin{array}{ccccc} 1 & -1 & 0 & \ldots & 0\\ 1 & 0 & -1 & \ldots & 0\\ \vdots & \vdots & \vdots & \ddots & \vdots \\ 1 & 0 & 0 & \ldots & -1 \end{array}\right).$$

Let V denote the covariance matrix of $\hat{\theta }$ and $\hat{\text{V}}$ its estimate. This is a submatrix of the covariance matrix of the parameters of the SCCS model for event‐dependent exposures. The Wald test statistic is then

$$X^{2}={\left(\text{H}\hat{\theta }\right)}^{T}{\left(\text{H}\hat{\text{V}}{\text{H}}^{T}\right)}^{-1}\text{H}\hat{\theta }.$$

As the sample size increases, $X^{2}$ converges in distribution to the chi‐squared density on k−1 degrees of freedom; this is a consequence of Slutsky's Theorem. When k=2, $X^{2}$ reduces to the test statistic described in Section 3.3.

The test may also be adapted to test equality of more than one relative incidence at each dose, for relative incidences corresponding to several risk periods. All that is needed is to alter the matrix of linear contrasts H so as to yield the contrasts corresponding to the hull hypothesis of interest.

The SCCS model for event‐dependent exposures may be fitted with function eventdepenexp from the R package SCCS. In version 1.4 of the package, the sandwich estimate of the covariance matrix may be extracted from the fitted SCCS model using the R command modelname$VarCov. This yields the estimate $\hat{\text{V}}$.
