# Supplementary material for: A modified self‐controlled case series method for event‐dependent exposures and high event‐related mortality, with application to COVID‐19 vaccine safety
Source: Stat Med. 2022 Jan 28;41(10):1735–50. doi: 10.1002/sim.9325 (PMC9303905; doi:10.1002/sim.9325)
Supplement: Supplementary file 1 — Table S1. Bias and mean squared error of the log relative incidence θ=log(ρ) at dose 1 (Monte Carlo standard error) when a proportion p of cases die of causes unrelated to the event, for selected values of p and ρ. Table S2. Bias and mean squared error of the log relative incidence θ=log(ρ) at dose 2 (Monte Carlo standard error) when a proportion p of cases die of causes unrelated to the event, for selected values of p and ρ. Table S3. Bias and mean squared error of the log relative incidence θ=log(ρ) at dose 1 (Monte Carlo standard error) for models including all cases and for models including only cases with events post‐vaccination, for selected values of ρ. Table S4. Bias and mean squared error of the log relative incidence θ=log(ρ) at dose 2 (Monte Carlo standard error) for models including all cases and for models including only cases with events post‐vaccination, for selected values of ρ. [file SIM-41-1735-s002.pdf]

A modified self-controlled case series method for event-dependent exposures and high event-related mortality, with application to COVID-19 vaccine safety

Supplementary Tables

Yonas Ghebremichael-Weldeselassie, Marie Joëlle Jabagi,
Jérémie Botton, Marion Bertrand, Bérangère Baricault,
Jérôme Drouin, Alain Weill, Mahmoud Zureik,
Rosemary Dray-Spira, Paddy Farrington

Supplementary Table 1: Bias and mean squared error of the log relative incidence $\theta = \log(\rho)$ at dose 1 (Monte Carlo standard error) when a proportion p of cases die of causes unrelated to the event, for selected values of p and ρ.

Proportion p	$\rho = 1$	$\rho = 2$	$\rho = 3$	$\rho = 4$
$p = 0$				
Bias	-0.0016 (0.0032)	-0.0009 (0.0030)	0.0014 (0.0029)	0.0035 (0.0029)
MSE	0.0103 (0.0005)	0.0088 (0.0004)	0.0087 (0.0004)	0.0084 (0.0004)
$p = 0.1$				
Bias	-0.0075 (0.0034)	-0.0184 (0.0030)	-0.0249 (0.0029)	-0.0298 (0.0029)
MSE	0.0113 (0.0005)	0.0094 (0.0004)	0.0088 (0.0004)	0.0093 (0.0004)
$p = 0.2$				
Bias	-0.0127 (0.0034)	-0.0279 (0.0031)	-0.0344 (0.0029)	-0.0560 (0.0029)
MSE	0.0115 (0.0005)	0.0104 (0.0005)	0.0096 (0.0004)	0.0117 (0.0005)
$p = 0.3$				
Bias	-0.0117 (0.0034)	-0.0355 (0.0030)	-0.0598 (0.0030)	-0.0714 (0.0031)
MSE	0.0117 (0.0005)	0.0103 (0.0005)	0.0128 (0.0006)	0.0148 (0.0006)

Supplementary Table 2: Bias and mean squared error of the log relative incidence $\theta = \log(\rho)$ at dose 2 (Monte Carlo standard error) when a proportion p of cases die of causes unrelated to the event, for selected values of p and ρ.

Proportion p	$\rho = 1$	$\rho = 2$	$\rho = 3$	$\rho = 4$
$p = 0$				
Bias	-0.0095 (0.0048)	-0.0038 (0.0046)	0.0063 (0.0047)	-0.0049 (0.0047)
MSE	0.0233 (0.0010)	0.0207 (0.0010)	0.0220 (0.0011)	0.0221 (0.0010)
$p = 0.1$				
Bias	-0.0005 (0.0049)	-0.0017 (0.0046)	-0.0090 (0.0045)	-0.0104 (0.0049)
MSE	0.0238 (0.0011)	0.0209 (0.0009)	0.0205 (0.0009)	0.0242 (0.0011)
$p = 0.2$				
Bias	0.0061 (0.0051)	-0.0111 (0.0045)	-0.0084 (0.0048)	-0.0116 (0.0048)
MSE	0.0258 (0.0011)	0.0200 (0.0009)	0.0232 (0.0010)	0.0232 (0.0010)
$p = 0.3$				
Bias	0.0030 (0.0053)	0.0044 (0.0048)	-0.0115 (0.0049)	-0.0238 (0.0051)
MSE	0.0276 (0.0012)	0.0232 (0.0011)	0.0240 (0.0012)	0.0262 (0.0011)

Supplementary Table 3: Bias and mean squared error of the log relative incidence $\theta = \log(\rho)$ at dose 1 (Monte Carlo standard error) for models including all cases and for models including only cases with events post-vaccination, for selected values of ρ.

Model	$\rho = 1$	$\rho = 2$	$\rho = 3$	$\rho = 4$
All cases included				
Bias	0.0014 (0.0032)	0.0050 (0.0029)	0.0018 (0.0029)	0.0003 (0.0029)
MSE	0.0101 (0.0005)	0.0086 (0.0004)	0.0083 (0.0004)	0.0086 (0.0004)
Post-vaccination cases only				
Bias	0.5637 (0.0040)	0.6012 (0.0039)	0.6115 (0.0039)	0.6219 (0.0040)
MSE	0.3337 (0.0046)	0.3764 (0.0047)	0.3893 (0.0049)	0.4028 (0.0050)

Supplementary Table 4: Bias and mean squared error of the log relative incidence $\theta = \log(\rho)$ at dose 2 (Monte Carlo standard error) for models including all cases and for models including only cases with events post-vaccination, for selected values of ρ.

Model	$\rho = 1$	$\rho = 2$	$\rho = 3$	$\rho = 4$
All cases included				
Bias	-0.0045 (0.0049)	0.0052 (0.0046)	0.0021 (0.0043)	0.0094 (0.0048)
MSE	0.0236 (0.0011)	0.0208 (0.0009)	0.0187 (0.0009)	0.0227 (0.0010)
Post-vaccination cases only				
Bias	0.2296 (0.0052)	0.2782 (0.0049)	0.2965 (0.0048)	0.3179 (0.0051)
MSE	0.0796 (0.0026)	0.1017 (0.0030)	0.1106 (0.0030)	0.1275 (0.0036)
